# Early Introduction of Severe Acute Respiratory Syndrome Coronavirus 2 into Europe

**DOI:** 10.3201/eid2607.200359

**Published:** 2020-07

**Authors:** Sonja J. Olsen, Meng-Yu Chen, Yu-Lun Liu, Mark Witschi, Alexis Ardoin, Clémentine Calba, Pauline Mathieu, Virginie Masserey, Francesco Maraglino, Stefano Marro, Pasi Penttinen, Emmanuel Robesyn, Jukka Pukkila, the European COVID-19 Work Group

**Affiliations:** World Health Organization Regional Office for Europe, Copenhagen, Denmark (S.J. Olsen, J. Pukkila);; Taiwanese Centers for Disease Control, Taipei (M.-Y. Chen, Y.-L. Liu);; Swiss Federal Office of Public Health, Bern, Switzerland (M. Witschi, V. Masserey);; Agence Régionale de Santé Ile-de-France, Paris, France (A. Ardoin); Santé Publique France, Paris (C. Calba);; Direction Générale de la Santé, Paris (P. Mathieu); Ministry of Health, Rome, Italy (F. Maraglino, S. Marro); European Centre for Disease Prevention and Control, Stockholm, Sweden (P. Penttinen, E. Robesyn)

**Keywords:** severe acute respiratory syndrome coronavirus 2, SARS-CoV-2, coronavirus, viruses, 2019 novel coronavirus disease, COVID-19, travel-related illness, transmission, respiratory infections, zoonoses, Europe

## Abstract

Early infections with severe acute respiratory syndrome coronavirus 2 in Europe were detected in travelers from Wuhan, China, in January 2020. In 1 tour group, 5 of 30 members were ill; 3 cases were laboratory confirmed. In addition, a healthcare worker was infected. This event documents early importation and subsequent spread of the virus in Europe.

A novel coronavirus, severe acute respiratory syndrome coronavirus 2 (SARS-CoV-2), associated with severe respiratory illness emerged in Wuhan, China, in late 2019 (*1*). Epidemiologic data indicate that the virus can cause a wide spectrum of clinical disease (mild-to-severe illness), including death (*2*–*4*), and spreads through direct contact and droplets.

Estimates are 5–6 days (range 2–14 days) for the incubation period and 2.2–3.6 for the reproduction rate; this rate is higher than those for seasonal and pandemic influenza (*5*,*6*). Extensive control efforts are now in place as part of a global containment strategy to minimize exportation from China and rapidly identify and stop international spread.

In the World Health Organization European Region, Rome, Paris, London, Istanbul, and Moscow have direct flights to Wuhan, China, and the risk for importation was considered high (*7*). SARS-CoV-2 was reported to have been introduced into Europe by a person from France who had traveled to Wuhan, China, for work, became ill on January 16, and returned ill to France on January 22 (*8*). We report a cluster of illness in a tour group from Wuhan that predates this case detection and led to subsequent transmission in Europe.

## The Study

A 55-year-old woman (Taiwanese tour guide) who resided in Wuhan came to airport health authorities in Taipei on January 25, 2020, complaining of a cough since January 22. She was transported to a designated hospital and showed a PCR-positive result for SARS-CoV-2 on January 26. She indicated that she had led a group of tourists from Wuhan to Europe on January 16–24. Further interviews with her and discussions with the rest of the group through social media yielded detailed information.

A group of 30 persons departed Wuhan on January 16, 2020, for a 9-day tour in Italy, Switzerland, and France (Table; Figure). During the flight on January 16 from Wuhan to Rome, 1 tour member was mildly ill and coughing. Her daughter became ill during the tour on January 21.

**Table:** Characteristics of 6 persons with suspected or confirmed cases of infection with severe acute respiratory syndrome coronavirus 2 associated with transmission in a tour group from Wuhan, China, to Europe, January–February, 2020*

Person	Age, y/sex	Onset of symptoms	Symptoms	Date of test result (specimen type)	Days between onset and test	Date of test result	Hospitalization date
Mother	53/F	Before Jan 16	Cough, no fever, odynophagia (cough still noted on return flight to China)	Not tested	NA	NA	None
Daughter	29/F	Jan 21	Cough, no fever, odynophagia (cough still noted on return flight to China)	Not tested	NA	NA	None
Tour guide	55/F	Jan 22	Dry cough, fever (Jan 26–Feb 2, Feb 10)	Jan 25 (throat and sputum)	3	Jan 26	Jan 25
Tour member	31/F	Jan 24	Diarrhea (Jan 24), cough (Jan 25), fever (Jan 31–Feb 3), facial palsy (Feb 3)	Jan 31 (throat swab)	7	Feb 1	Jan 31
Tour leader	27/F	Jan 25	Febrile sensation (low-grade fever of 37.4°C)	Feb 1 (throat swab)	7	Feb 3	Feb 5
Physician	53/M	Jan 28	Fever, headache, general weakness	Jan 29, first positive; Feb 2–4, daily positive; Feb 5–9, daily positive (low); Feb 11–14, daily negative	1	Jan 29	Jan 29

*NA, not applicable.

**Figure:**
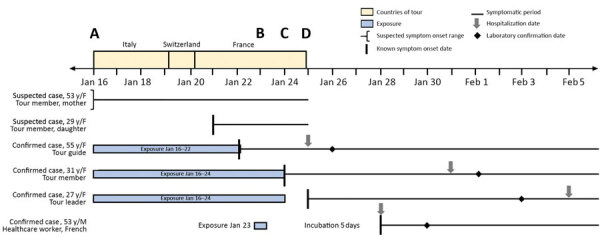
Timeline showing illness onsets and exposures for 6 persons with suspected or confirmed cases of infection with severe acute respiratory syndrome coronavirus 2 associated with transmission in a tour group flying from Wuhan, China, to Europe, January–February, 2020. A) Flight from Wuhan to Rome; B) 2 case-patients visited by healthcare worker; C) return flight from Paris to Guangdong; D) tour guide flight to Taipei.

On January 23, while in Paris, the mother and daughter decided to seek medical care. They called the Chinese embassy, who told them to call the emergency hotline (at SAMU Centre 15 Hospital, Paris, France) dedicated to evaluation of suspected 2019 novel coronavirus disease (COVID-19) cases in France (*8*). The emergency hotline routed the call to the 24-hour ambulatory service, but no information about suspicion of COVID-19 was given. A physician came to their hotel room and gave them a diagnosis of the common cold. The interaction lasted ≈20 min, including a 15-minute face-to-face examination, without protective masks for the patients or any personal protective equipment for the physician. Also present was another member of the group who translated. While in Paris, the 3 ill persons bought surgical face masks and began wearing them on January 21 (mother and daughter) and on January 22 (tour guide).

The tour group departed on January 24 from Paris to Guangzhou, China (arrival on January 25), because Wuhan ceased air traffic on January 23. The tour guide subsequently continued from Guangzhou to Taipei on January 25, where she arrived on January 25 and was hospitalized. After returning to China, 2 additional tour members, including the tour leader, reported illness and were hospitalized in Hubei and Jiangsu Provinces, where they showed positive test results for SARS-CoV-2. Five (17%) of the 30 tour members were ill; 3 had laboratory-confirmed infection, and 2 were never tested. Because of prolonged and overlapping exposure of the group, it is impossible to determine the exact source of all infections. The source for the physician could have been the ill suspected case-patients, whose infections were never confirmed, or the then-presymptomatic person who translated (contact was 2 days before illness onset).

The tour group was a fairly contained group that did not have much prolonged contact with others. Other than the 3 ill persons, the group members did not wear masks during the tour. 

As part of the investigation, the countries the tour visited identified low-risk (<15 min, >1–2 m) and high-risk (>15 min, <1–2 m) contacts (*8*). Most transport was made by using 1 bus, except for 1 section of travel by train in Switzerland. The bus driver, from Slovakia, was a high-risk contact; he returned to Slovakia after the tour and denied having any symptoms in the 14 days after last contact with the tour group. 

In Italy, because seat numbers on the flight from Wuhan to Rome, during which 1 tour group member had a cough, were not reported in the passengers list, authorities contacted and informed all passengers (n = 176) and crew members (n = 17). The information provided was to watch for development of symptoms and call if any developed. No other high-risk contacts of the symptomatic group member in Italy were identified.

In Switzerland, health authorities identified 0 high-risk and 3 low-risk contacts, (1 restaurant owner and 2 shop clerks). These 3 persons were told to watch for development of symptoms and call if any developed. None were reported.

In France, the group visited several tourist attractions and used public transportation; contacts in the shops and hotel were interviewed and defined as low-risk contacts. Only 1 high-risk contact was identified. This person was the physician.

The physician was not wearing a mask during the consultation because he had not been informed of the risk of COVID-19. He became ill on January 28 and stopped seeing patients. He went to a designated referral hospital on January 29 and showed a positive PCR result for SARS-CoV-2 for 12 days; he has since recovered. A total of 58 contacts (20 low risk and 38 high risk) of the physician were identified, including patients and their family members he saw during home visits the day before onset of symptoms. All involved persons have been investigated and followed-up for 14 days; none showed development of illness. 

Authorities in China were notified of symptomatic persons on the flight from Paris to Guangzhou. However, we were unable to confirm whether any contact tracing was performed.

As of February 10, the tour guide remained hospitalized, but had a normal chest radiograph and was clinically well. She remains in isolation because of a virus-positive sputum test result 19 days after illness onset. The other 3 tour members who were ill reportedly experienced mild illness, and all are now well. Because the first 2 symptomatic persons were never tested, we cannot conclude that they were the source of infection. However, given that the virus was not circulating in France, the source was most likely in the tour group. It is also possible that additional transmission resulting in mild illness occurred, particularly in the tour group, but was not identified.

## Conclusions

This event represents early introduction of SARS-CoV-2 into Europe, before implementation of extensive travel restrictions in Wuhan on January 23, and could explain additional chains of transmission in France, where the disease has now spread widely. The event was characterized by clinically mild illness in 6 persons; 2 showed documented prolonged virus shedding. Excluding members of the tour group, 1 of 40 high-risk and 0 of 216 low-risk contacts became ill. The 1 high-risk exposure event was short but entailed close contact during a clinical examination. Assuming this was the sole exposure, the incubation time was 5 days, which is consistent with reported data.

This event represented a coordinated international effort and highlights the effectiveness of working through the established mechanisms of the European Union Early Warning and Response System and the International Health Regulations. This effort will be key to the effective implementation of the current global containment strategy.
